# Crystal structures of three platinacyclic complexes bearing isopropyl eugenoxyacetate and pyridine derivatives

**DOI:** 10.1107/S2056989020006957

**Published:** 2020-06-05

**Authors:** Nguyen Thi Thanh Chi, Pham Van Thong, Luc Van Meervelt

**Affiliations:** a Department of Chemistry, Hanoi National University of Education, 136 Xuan Thuy, Cau Giay, Hanoi, Vietnam; b Department of Chemistry, KU Leuven, Biomolecular Architecture, Celestijnenlaan 200F, Leuven (Heverlee), B-3001, Belgium

**Keywords:** crystal structure, platinum(II) complexes, eugenol, pyridine derivatives, cytotoxicity

## Abstract

Three new platinum(II) complexes bearing a eugenol and a pyridine derivative, namely (η^2^-2-allyl-4-meth­oxy-5-{[(propan-2-yl­oxy)carbon­yl]meth­oxy}phenyl-κ*C* ^1^)chlorido­(pyridine-κ*N*)platinum(II), [Pt(C_15_H_19_O_4_)Cl(C_5_H_5_N)], (**I**), (η^2^-2-allyl-4-meth­oxy-5-{[(propan-2-yl­oxy)carbon­yl]meth­oxy}phenyl-κ*C* ^1^)chlorido­(4-methyl­pyridine-κ*N*)platinum(II), [Pt(C_15_H_19_O_4_)Cl(C_6_H_7_N)], (**II**), and (η^2^-2-allyl-4-meth­oxy-5-{[(propan-2-yl­oxy)carbon­yl]meth­oxy}phenyl-κ*C* ^1^)chlorido(pyridine-4-carb­oxy­lic acid-κ*N*)platinum(II), [Pt(C_15_H_19_O_4_)Cl(C_6_H_5_NO_2_)], (**III**), have been synthesized and further characterized by single-crystal X-ray diffraction. The Pt^II^ atoms exhibit the usual distorted square-planar coordination and are surrounded by one Cl atom, one N atom, and a C atom and C=C double bond of the eugenol ligand. The donor N atom of the pyridine ligand occupies a *cis* position with respect to the double bond. Complexes (**I**) and (**II**) crystallize isomorphously in space group *P*  and display a similar crystal packing characterized by C—H⋯O hydrogen bonding, C—H⋯π and π–π inter­actions. However, the presence of the additional methyl group in the 4-methyl­pyridine ligand in (**II**) disturbs the π–π inter­actions. The crystal packing of (**III**) is characterized by O—H⋯O hydrogen bonding, resulting in the formation of chains of mol­ecules connected in a head-to-tail fashion and running in the [101] direction. The IC_50_ values for the HepG2 and KB cell lines are 150.9, 122.3 µ*M* for (**I**) and 138.9, 93.2 µ*M* for (**II**), respectively.

## Abstract 2

Three new platinum(II) complexes bearing an isopropyl eugenoxyacetate and pyridine derivatives have been synthesized and further characterized by single-crystal X-ray diffraction.

## Chemical context   

Although platinum-based drugs have dominated the treatment of various cancers by chemical agents, the research on new platinum(ll) complexes for the purpose of medical application is still attractive for the worldwide scientific society (Johnstone *et al.*, 2016 ▸). Recently, numerous platinum(ll) complexes bearing alkene and pyridine derivatives have been synthesized and tested for their anti-cancer activities (Bigioni *et al.*, 2000 ▸; Da *et al.*, 2012 ▸, 2015 ▸; Chi *et al.*, 2017 ▸, 2018 ▸; Cucciolito *et al.*, 2018 ▸; Dodoff *et al.*, 2012 ▸). Nevertheless, crystal data for these complexes are limited, some examples being the crystal structures of [PtCl(eugenol-1*H*)(pyridine)], [PtCl(eugenol-1*H*)(4-methyl­pyridine)] (Chi *et al.*, 2018 ▸) and *trans*-[PtCl_2_(C_2_H_4_)(*N*-3-pyridinyl­methane­sulfonamide)] (Do­doff *et al.*, 2012 ▸).

**Figure 1:**
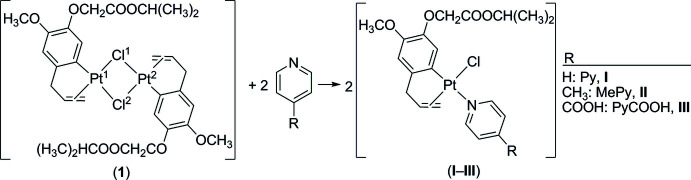
Reaction scheme for the synthesis of mixed ^*i*^PrEug-pyridine derivative platinum(II) complexes (**I**), (**II**) and (**III**).

In this paper, the crystal structures of three mononuclear platinacyclic complexes namely, (η^2^-2-allyl-4-meth­oxy-5-{[(propan-2-yl­oxy)carbon­yl]meth­oxy}phenyl-κ*C* ^1^)chlorido(pyridine-κ*N*)platinum(II), [Pt(C_15_H_19_O_4_)Cl(C_5_H_5_N)], (**I**), (η^2^-2-allyl-4-meth­oxy-5-{[(propan-2-yl­oxy)carbon­yl]methoxy}phenyl-κ*C* ^1^)chlorido­(4-methyl­pyridine-κ*N*)platinum(II), [Pt(C_15_H_19_O_4_)Cl(C_6_H_7_N)], (**II**), and (η^2^-2-allyl-4-meth­oxy-5-{[(propan-2-yl­oxy)carbon­yl]meth­oxy}phenyl-κ*C* ^1^)chlorido(pyridine-4-carb­oxy­lic acid-κ*N*)platinum(II), [Pt(C_15_H_19_O_4_)Cl(C_6_H_5_NO_2_)], (**III**), are reported. Complexes (**I**), (**II**), (**III**) are obtained from the reactions of the dinuclear chelate ring complex [Pt(*μ*-Cl)(^*i*^PrEug)]_2_ (**1**, ^*i*^PrEug: deprotonated isopropyl eugenoxyacetate) with pyridine (Py), 4-methyl­pyridine (MePy) and pyridine-4-carb­oxy­lic acid (PyCOOH), respectively. The synthesis of the three complexes is summarized in Fig. 1 ▸.
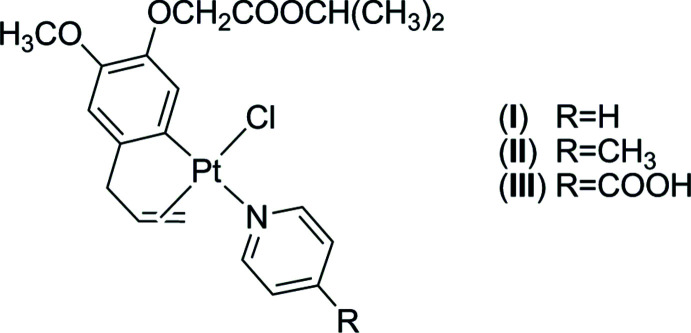


The Py, MePy and PyCOOH cleave the Pt^1^—Cl^2^ (or Pt^2^—Cl^1^) bond in complex **1** to form complexes (**I**), (**II**), (**III**). This is due to the weaker Pt^1^—Cl^2^ or Pt^2^—Cl^1^ bond (2.4773 Å) as compared to the Pt^1^—Cl^1^ or Pt^2^—Cl^2^ bond (2.3527 Å) (Nguyen Thi Thanh *et al.*, 2016 ▸) and results in a *cis* but not *trans* position of the pyridine ligands with respect to the allyl group of ^*i*^PrEug. Similar results have been observed when the complexes [Pt(*μ*-Cl)(aryl­olefin-1H)]_2_ (aryl­olefin: safrole or eugenol derivatives) analogous to **1** react with different amines (Da *et al.*, 2012 ▸, 2015 ▸; Chi *et al.*, 2018 ▸).

## Structural commentary   

**Figure 2:**
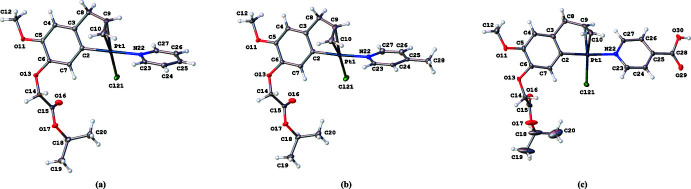
The mol­ecular structure of complexes (**I**), (**II**) and (**III**) showing the atom-labelling scheme and displacement ellipsoids at the 50% probability level.

**Figure 3:**
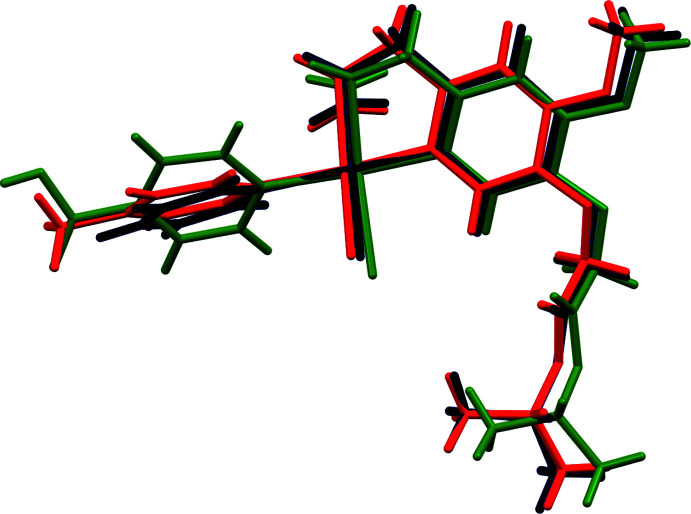
Overlay of the three complexes, showing the different conformation of the pyridine ring for (**III**). Complex (**I**) is in black, complex (**II**) in red and complex (**III**) in green.

Complexes (**I**) and (**II**) crystallize isomorphously in the triclinic space group *P* 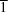. The central Pt^II^ atom displays a distorted square-planar coordination with the Cl atom, the N atom of the pyridine or 4-methyl­pyridine ligand, and completed with a C atom and C=C double bond of the eugenol ligand (Fig. 2 ▸ *a* and 2*b*). The C=C group and N atom are in a *cis* position with respect to each other. The dihedral angle between the best planes through the pyridine and phenyl rings is 74.90 (15)° for complex (**I**) and 75.00 (11)° for complex (**II**). The dihedral angle between the planes through the allyl atoms (C8, C9, C10) and the pyridine ring is 16.0 (2)° for complex (**I**) and 20.08 (12)° for complex (**II**). The almost identical conformation is further evidenced by a fit of both structures, excluding H atoms and the methyl substituent in (**II**), which gives an r.m.s. deviation of 0.1867 Å (Fig. 3 ▸).

Complex (**III**) also crystallizes in space group *P* 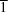, but due to the presence of the carb­oxy­lic acid function the crystal structure is no longer isomorphous with (**I**) and (**II**) (Fig. 2 ▸ *c*). Although the square-planar coordination of the central Pt^II^ atom is identical, the dihedral angle of 21.6 (2)° illustrates that the mutual orientation of the eugenol and pyridine parts is different. The plane through the allyl group makes an angle of 40.9 (3)° with the pyridine plane. An overlay of the identical parts in (**I**) and (**III**) gives an r.m.s. deviation of 0.5782 Å, while 0.5507 Å for (**II**) and (**III**) (Fig. 3 ▸).

Comparing the bond distances in the coordination sphere of the central Pt^II^ atom of the three complexes shows that the largest differences occur for the Pt—N distance: 2.139 (2) Å for (**I**) within experimental error the same as 2.1418 (18) Å for (**II**), and 2.164 (3) Å for (**III**).

## Supra­molecular features   

**Figure 4:**
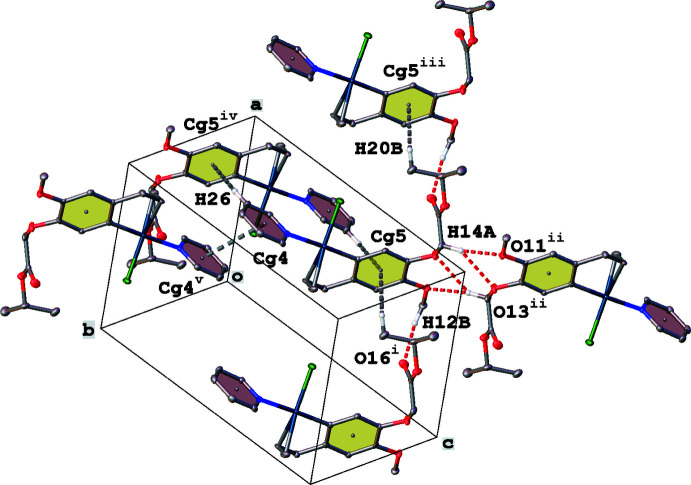
Partial crystal packing of complex (**I**), showing C—H⋯O hydrogen bonding (red dashed lines), C—H⋯π and π–π inter­actions (grey dashed lines). Hydrogen atoms not involved in inter­actions have been omitted for clarity (see Table 1 ▸ for symmetry codes).

The crystal packing of complex (**I**) is characterized by C—H⋯O hydrogen bonding, C—H⋯π and π–π inter­actions (Fig. 4 ▸, Table 1 ▸). The bifurcated hydrogen bond between C14—H14*A* and O11/O13 gives rise to the formation of inversion dimers. The eugenol parts are further linked into chains running in the *a*-axis direction by C12—H12*B*⋯O16 hydrogen-bond inter­actions. Further dimer formation is obtained through π–π stacking between the pyridine rings [*Cg*4⋯*Cg*4^v^ = 3.560 (2) Å; *Cg*4 is the centroid of ring N22/C23–C27; symmetry code: (v) 2 − *x*, 2 − *y*, 1 − *z*]. The phenyl ring C2–C7 participates in two C—H⋯π inter­actions.

**Figure 5:**
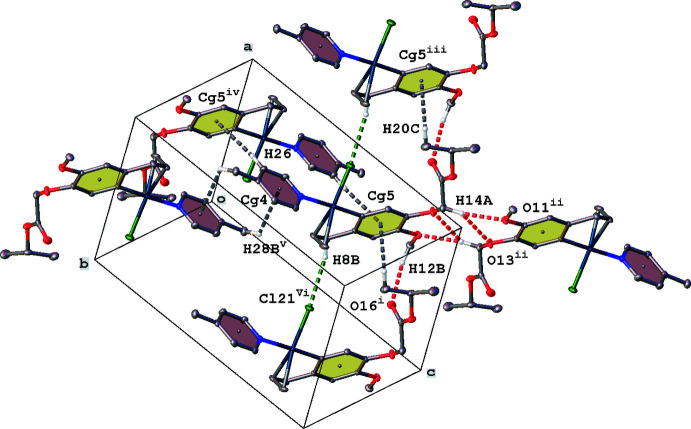
Partial crystal packing of complex (**II**), showing C—H⋯O hydrogen bonding (red dashed lines), C—H⋯Cl (green dashed lines), C—H⋯π and π–π inter­actions (grey dashed lines). Hydrogen atoms not involved in inter­actions have been omitted for clarity (see Table 2 ▸ for symmetry codes).

Complex (**II**) displays a very similar crystal packing (Fig. 5 ▸, Table 2 ▸). But, due to the presence of a 4-methyl­pyridine ring in (**II**), the π–π stacking is absent [*Cg*4⋯*Cg*4^v^ = 4.312 (1) Å, slippage 2.703 Å; *Cg*4 is the centroid of ring N22/C23–C27; symmetry code: (v) 2 − *x*, 2 − *y*, 1 − *z*] and is in fact replaced by two C—H⋯π inter­actions between the methyl group and the pyridine ring. This slippage of the pyridine ring also results in an additional C8—H8*B*⋯Cl21 inter­action between the allyl CH_2_ group and a neighboring Cl atom.

**Figure 6:**
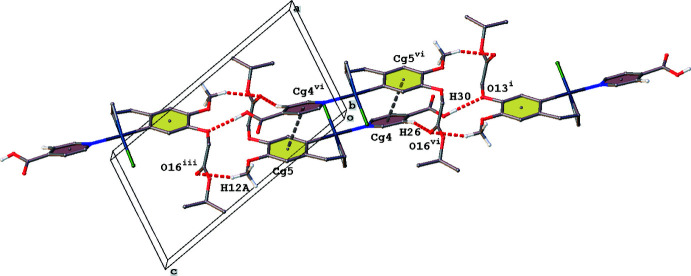
Partial crystal packing of complex (**III**), showing the chain formation in the [101] direction. O—H⋯O and C—H⋯O hydrogen bonding are shown as red dashed lines, π–π inter­actions as grey dashed lines. Hydrogen atoms not involved in inter­actions have been omitted for clarity (see Table 3 ▸ for symmetry codes).

The carb­oxy­lic acid function present in complex (**III**) is involved in head-to-tail fashion O—H⋯O inter­actions resulting in the formation of chains running in the [101] direction (Fig. 6 ▸, Table 3 ▸). Parallel chains inter­act through π–π inter­actions [*Cg*4⋯*Cg*5^vi^ = 3.947 (2) Å; *Cg*4 and *Cg*5 are the centroids of rings N22/C23–C27 and C2–C7, respectively; symmetry code: (vi) −*x*, 1 − *y*, −*z*] and C—H⋯O hydrogen-bonding inter­actions (Fig. 6 ▸, Table 3 ▸).

No voids are observed in the crystal packing of complexes (**I**) and (**II**), but for complex (**III**) a small void of 37 Å^3^ is present around (½, 0, 0).

## Database survey   

A search of the Cambridge Structural Database (CSD, Version 5.41, update of November 2019; Groom *et al.*, 2016 ▸) for Pt complexes with the Pt atom coordinated to a Cl atom, N atom and allylaryl ligand (similar to the title complexes) gave eight hits. The C=C group and N atom are always in a *cis* position with respect to each other. All complexes also possess a distorted square-planar coordination for the Pt atom with a deviation of the Pt atom from the best plane through the coordinating Cl, N, C_ar­yl_ and centroid (*Cg*) of the C=C group between 0.018 Å [chloro-(4,5-dimeth­oxy-2-prop-2-en-1-yl)phenyl-(2-methyl­aniline)platinum(II), refcode GOY­JEL; Da *et al.*, 2015 ▸] and 0.048 Å [(η^2^-5-hy­droxy-4-meth­oxy-2-(prop-2-en-1-yl)phen­yl)-chloro-(4-methyl­pyridine)­platin­um(II), CSD refcode VEZJIW; Chi *et al.*, 2018 ▸]. Table 4 ▸ gives an overview of the four Pt bond distances for each compound. The average Pt—Cl, Pt—N, Pt—C_ar­yl_ and Pt—*Cg* distances are 2.324 (8), 2.158 (29), 1.996 (64) and 2.014 (16) Å, respectively. The largest spread is observed for the Pt—C_ar­yl_ bond (1.843 to 2.109 Å in the two mol­ecules present in the asymmetric unit of chloro-(η^2^-6-ethenyl-1,3-benzodioxole-5-yl)piperidine­platinum(II) (CSD refcode OFUREN; Da *et al.*, 2008 ▸). The averages correspond to the observed distances for complexes (**I**)–(**III**). It is worthwhile to note that upon binding to Pt, the C=C bond distance [1.29 (4) Å for allylaryl fragments in the CSD] increased significantly. The average C=C bond distance for the complexes in Table 4 ▸ is 1.39 (3) Å, comparable to the C=C bond distances in the title complexes [1.389 (4), 1.401 (3) and 1.376 (6) Å for (**I**)–(**III**), respectively].

## *In vitro* cytotoxicity of complexes (I) and (II)   

The *in vitro* cytotoxicity of complexes (**I**) and (**II**) was tested according to the method described in Skehan *et al.* (1990 ▸) and Likhitwitayawuid *et al.* (1993 ▸) on two human cancer cell lines of HepG2 (hepatocellular carcinoma) and KB (human epidermal carcinoma). The IC_50_ values for the HepG2 and KB cell lines calculated based on OD values taken on an Elisa instrument at 515–540 nm are 150.9, 122.3 µ*M* for (**I**) and 138.9, 93.2 µ*M* for (**II**), respectively. This result shows that the presence of the extra methyl group on the pyridine ring in the *para* position in (**II**) does not have a notable effect on its anti-cancer activities as compared to those of (**I**). However, a comparison of complexes that differ solely in the olefin ligand reveals a significant influence. Specifically, complex (**I**) exhibits much better cytotoxicity against HepG2 and KB cell lines than [PtCl(eugenol-1H)(Py)] (>270.7, 211.8 µ*M*, respectively; Chi *et al.*, 2018 ▸) but worse than [Pt(methyl­eugenol-1H)(Py)] (7.07 µ*M* for KB cell line; Da *et al.*, 2015 ▸).

## Synthesis and crystallization   

The synthetic protocol for the three complexes is shown in Fig. 1 ▸. The starting complex [Pt(*μ*-Cl)(^*i*^PrEug)]_2_ (**1**) was synthesized according to the synthetic protocol of Thong & Chi (2014 ▸).

**[PtCl(** ***^i^*** **PrEug)(pyridine)]** (**I**). A solution of pyridine (80 µL, 1.0 mmol) in 10 mL ethanol was slowly added with stirring to a suspension of [Pt(*μ*-Cl)(^*i*^PrEug)]_2_ (494 mg, 0.5 mmol) in 10 mL acetone. The reaction mixture was stirred at ambient temperature (AT) and filtered off after 30 minutes to remove the insoluble part. Subsequently, slow evaporation of the solvent of the obtained solution at AT gave within 10 h transparent crystals, which were suitable for X-ray diffraction and other analyses. The yield was 515 mg (90%). %Pt (found/calculated): 34.15/34.06. ESI MS (*m/z*, intensity), −MS: 1021, 100%, [2*M* – 2Py + Cl]^−^; +MS: 1067, 100%, [2*M* – Py + H]^+^; 988, 30%, [2*M* – 2Py + H]^+^. IR (cm^−1^, ν): 3089, 2970 and 2839 (CH); 1748 (C=O); 1597 and 1477 (C=C, C=N). ^1^H NMR (500 MHz, acetone-*d_6_*): 8.79 (*ov*, 2H, Ar—H), 8.04 (*m*, 1H, Ar—H), 7.65 (*ov*, 2H, Ar—H), 7.04 (*s*, ^3^ *J*_PtH_ = 40, 1H, Ar—H), 6.66 (*s*, 1H, Ar—H), 5.07 (*m*, 1H, O—CH), 4.83 (*m*, ^2^ *J*_PtH_ = 70 Hz, 1H, C*H*=CH_2_), 4.54 (*s*, 2H, OCH_2_), 3.81 [*d*, ^3^ *J*(H,H) = 13.0 Hz, 1H, CH=C*H*_2_], 3.78–3.74 (*ov*, 2H, CH=C*H*_2_, C*H*_2_—CH), 3.73 (*s*, 3H, OCH_3_), 2.66 (*d*, ^2^ *J*(H,H) = 16.5 Hz, ^3^ *J*_PtH_ = 110 Hz, 1H, C*H*_2_—CH), 1.27 [*d*, ^3^ *J*(H,H) = 6.5 Hz, 6H, CH—(C*H*_3_)_2_].

**[PtCl(** ***^i^*** **PrEug)(4-methyl­pyridine)]** (**II**). This complex was prepared starting from [Pt(*μ*-Cl)(^*i*^PrEug)]_2_ (494 mg, 0.5 mmol) and 4-methyl­pyridine (100 µL, 1.0 mmol) according to the procedure for the synthesis of **I**. The yield was 539 mg (92%), transparent crystals were suitable for X-ray diffraction and other analyses. %Pt (found/calculated): 32.34/32.25. ESI MS (*m/z*, intensity), −MS: 1021, 100%, [2*M* – 2MePy + Cl]^−^; +MS: 1079, 70%, [2*M* – MePy + H]^+^; 986, 25%, [2*M* – 2MePy + H]^+^;. IR (cm^−1^, ν): 2970, 2920 and 2839 (CH); 1748 (C=O); 1616 and 1477 (C=C, C=N). ^1^H NMR (500 MHz, acetone-*d_6_*): 8.60 [*d*, ^3^ *J*(H,H) = 5.5 Hz, 2H, Ar—H], 7.46 [*d*, ^3^ *J*(H,H) = 5.5 Hz, 2H, Ar—H], 7.04 (*s*, ^3^ *J*_PtH_ = 40, 1H, Ar—H), 6.65 (*s*, 1H, Ar-H), 5.07 (*m*, 1H, O—CH), 4.79 (*m*, ^2^ *J*_PtH_ = 70 Hz, 1H, C*H*=CH_2_), 4.54 (*s*, 2H, OCH_2_), 3.78 [*d*, ^3^ *J*(H,H) = 13.0 Hz, 1H, CH=C*H*_2_], 3.76–3.74 (*ov*, 2H, CH=C*H*_2_, C*H*_2_—CH), 3.73 (*s*, 3H, OCH_3_), 2.64 [*d*, ^2^ *J*(H,H) = 16.5 Hz, 1H, C*H*_2_—CH], 2.46 (*s*, 3H, CH_3_), 1.27 [*d*, ^3^ *J*(H,H) = 6.5 Hz, 6H, CH—(C*H*_3_)_2_].

**[PtCl(** ***^i^*** **PrEug)(pyridine-4-carb­oxy­lic acid)]** (**III**). A mixture of pyridine-4-carb­oxy­lic acid (123 mg, 1.0 mmol) and [Pt(*μ*-Cl)(^*i*^PrEug)]_2_ (494 mg, 0.5 mmol) in 10 mL acetone was stirred at AT for 8 h. The resulting precipitate was filtered off and washed consecutively with ethanol (2 × 5 mL) and cold chloro­form (2 × 5 mL), then crystallized in chloro­form to give a light-yellow powder. The yield was 493 mg (80%). Single crystals suitable for X-ray diffraction were obtained by slow evaporation within 8 h from a concentrated chloro­form/ethanol solution at AT. %Pt (found/calculated): 31.58/31.63. ESI MS (*m/z*, intensity), -*M*S: 1021, 100%, [2*M* – 2PyCOOH + Cl]^−^; +MS: 1110, 8%, [2*M* – PyCOOH + H]^+^; 989, 10%, [2*M* – 2PyCOOH + H]^+^. IR (cm^−1^, ν): 3267 (OH), 3093, 2974 and 2839 (CH); 1728 (C=O); 1586 and 1477 (C=C, C=N). ^1^H NMR (500 MHz, dimethyl sulfoxide-*d_6_*): 13.80 (*br*, 1H, OH), 8.79 [*d*, ^3^ *J*(H,H) = 4.5 Hz, 2H, Ar—H], 7.83 [*d*, ^3^ *J*(H,H) = 4.5 Hz, 2H, Ar—H], 6.75–6.74 (*ov*, 2H, Ar—H), 5.08 (*m*, 1H, C*H*=CH_2_), 4.97 (*m*, 1H, O—CH), 4.58/4.51 [*d*, ^2^ *J*(H,H) = 16.5 Hz, 2H, OCH_2_], 4.33 [*d*, ^3^ *J*(H,H) = 6.0 Hz, 1H, CH=C*H*_2_], 3.93 [*d*, ^3^ *J*(H,H) = 13.5 Hz, 1H, CH=C*H*_2_], 3.79–3.70 (*ov*, 4H, C*H*_2_—CH, OCH_3_), 2.77 [*d*, ^2^ *J*(H,H) = 17.0 Hz, 1H, C*H*_2_—CH], 1.23 [*d*, ^3^ *J*(H,H) = 6.0 Hz, 6H, CH—(C*H*_3_)_2_].

## Refinement   

Crystal data, data collection and structure refinement details are summarized in Table 5 ▸.

The H atoms were placed in idealized positions and included as riding contributions with *U*_iso_(H) values of 1.2*U*_eq_ or 1.5*U*_eq_ of the parent atoms, with C—H distances of 0.95 (aromatic), 1.00 (CH), 0.99 (CH_2_) and 0.98 Å (CH_3_). The carb­oxy­lic acid H atom in (**III**) was refined as rotating group with a O—H distance of 0.84 Å. The displacement parameters of the bonded atoms in the carb­oxy­lic acid and isopropyl groups in (**III**) were restrained to be similar along the bond.

## Supplementary Material

Crystal structure: contains datablock(s) I, II, III. DOI: 10.1107/S2056989020006957/mw2159sup1.cif

Structure factors: contains datablock(s) I. DOI: 10.1107/S2056989020006957/mw2159Isup2.hkl

Structure factors: contains datablock(s) II. DOI: 10.1107/S2056989020006957/mw2159IIsup3.hkl

Structure factors: contains datablock(s) III. DOI: 10.1107/S2056989020006957/mw2159IIIsup4.hkl

CCDC references: 2005230, 2005229, 2005228

Additional supporting information:  crystallographic information; 3D view; checkCIF report

## Figures and Tables

**Table 1:** Hydrogen-bond geometry (Å, °) for (**I**) *Cg*5 is the centroid of the C2–C7 phenyl ring.

*D*—H⋯*A*	*D*—H	H⋯*A*	*D*⋯*A*	*D*—H⋯*A*
C12—H12*B*⋯O16^i^	0.98	2.42	3.354 (3)	160
C14—H14*A*⋯O11^ii^	0.99	2.31	3.266 (3)	161
C14—H14*A*⋯O13^ii^	0.99	2.56	3.330 (4)	134
C20—H20*B*⋯*Cg*5^iii^	0.98	2.93	3.586 (3)	125
C26—H26⋯*Cg*5^iv^	0.95	2.88	3.736 (3)	150

Symmetry codes: (i) 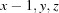; (ii) 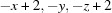; (iii) 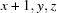; (iv) 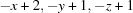.

**Table 2:** Hydrogen-bond geometry (Å, °) for (**II**) *Cg*5 is the centroid of the C2–C7 phenyl ring.

*D*—H⋯*A*	*D*—H	H⋯*A*	*D*⋯*A*	*D*—H⋯*A*
C12—H12*B*⋯O16^i^	0.98	2.45	3.397 (3)	162
C14—H14*A*⋯O11^ii^	0.99	2.39	3.341 (3)	161
C14—H14*A*⋯O13^ii^	0.99	2.57	3.351 (3)	136
C8—H8*B*⋯Cl21^i^	0.99	2.76	3.713 (3)	162
C20—H20*B*⋯*Cg*5^iii^	0.98	2.87	3.562 (3)	128
C26—H26⋯*Cg*5^iv^	0.95	2.93	3.873 (3)	171
C28—H28*B*⋯*Cg*4^v^	0.98	2.87	3.425 (3)	117

Symmetry codes: (i) 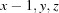; (ii) 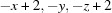; (iii) 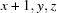; (iv) 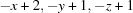; (v) 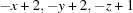.

**Table 3:** Hydrogen-bond geometry (Å, °) for (**III**)

*D*—H⋯*A*	*D*—H	H⋯*A*	*D*⋯*A*	*D*—H⋯*A*
O30—H30⋯O13^i^	0.84	2.10	2.932 (4)	170
C10—H10*A*⋯O16^ii^	0.95	2.41	3.317 (5)	159
C12—H12*A*⋯O16^iii^	0.98	2.51	3.415 (5)	154
C14—H14*A*⋯O29^iv^	0.99	2.46	3.268 (5)	139
C14—H14*B*⋯O29^v^	0.99	2.46	3.178 (5)	129
C26—H26⋯O16^vi^	0.95	2.43	3.336 (5)	159

Symmetry codes: (i) 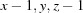; (ii) 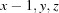; (iii) 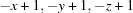; (iv) 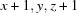; (v) 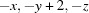; (vi) 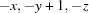.

**Table 4:** Pt bond distances (Å) for Pt complexes with the Pt atom coordinated to a Cl atom, N atom and allylaryl ligand found in the Cambridge Structural Database C_ar­yl_ is the aryl C atom and *Cg* the centroid of the C=C group of the coordinating allylaryl ligand.

CSD refcode	Pt—Cl	Pt—N	Pt—C_ar­yl_	Pt—*Cg*	Reference
EWAVOP	2.323	2.107	1.995	2.011	Nguyen Thi Thanh *et al.* (2016 ▸)
GOYJEL	2.324	2.177	2.001	2.011	Da *et al.* (2015 ▸)
OFUREN	2.319	2.160	2.109	2.057	Da *et al.* (2008 ▸)
OFUREN	2.340	2.187	1.843	1.995	Da *et al.* (2008 ▸)
SOMNUF	2.329	2.188	2.015	2.009	Mangwala Kimpende *et al.* (2014 ▸)
TALTIM	2.321	2.143	2.002	2.009	Le Thi Hong *et al.* (2017 ▸)
VEZHOA	2.332	2.140	2.006	2.010	Chi *et al.* (2018 ▸)
VEZJIW	2.314	2.142	1.991	2.007	Chi *et al.* (2018 ▸)
VEZJIW	2.318	2.138	1.999	2.017	Chi *et al.* (2018 ▸)
VEZJOC	2.317	2.199	2.002	2.015	Chi *et al.* (2018 ▸)

**Table 5:** Experimental details

	(**I**)	(**II**)	(**III**)
Crystal data			
Chemical formula	[Pt(C_15_H_19_O_4_)Cl(C_5_H_5_N)]	[Pt(C_15_H_19_O_4_)Cl(C_6_H_7_N)]	[Pt(C_15_H_19_O_4_)Cl(C_6_H_5_NO_2_)]
*M*_r_	572.94	586.97	616.95
Crystal system, space group	Triclinic, *P* 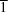	Triclinic, *P* 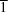	Triclinic, *P* 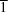
Temperature (K)	100	100	100
*a*, *b*, *c* (Å)	8.3146 (3), 8.6714 (4), 14.5827 (6)	8.36089 (15), 9.12717 (16), 14.5582 (3)	7.8746 (2), 9.7566 (2), 15.0004 (4)
α, β, γ (°)	90.534 (4), 104.376 (4), 101.135 (3)	94.9089 (15), 102.2766 (16), 100.4541 (15)	95.782 (2), 102.874 (2), 93.843 (2)
*V* (Å^3^)	997.49 (7)	1058.58 (3)	1113.02 (5)
*Z*	2	2	2
Radiation type	Mo *K*α	Mo *K*α	Mo *K*α
μ (mm^−1^)	7.19	6.78	6.46
Crystal size (mm)	0.25 × 0.2 × 0.15	0.25 × 0.2 × 0.2	0.4 × 0.4 × 0.35

Data collection			
Diffractometer	Rigaku Oxford Diffraction SuperNova, Single source at offset/far, Eos	Rigaku Oxford Diffraction SuperNova, Single source at offset/far, Eos	Rigaku Oxford Diffraction SuperNova, Single source at offset/far, Eos
Absorption correction	Multi-scan *CrysAlis PRO* (Rigaku OD, 2018 ▸)	Multi-scan *CrysAlis PRO* (Rigaku OD, 2018 ▸)	Multi-scan *CrysAlis PRO* (Rigaku OD, 2018 ▸)
*T*_min_, *T*_max_	0.717, 1.000	0.671, 1.000	0.429, 1.000
No. of measured, independent and observed [*I* > 2σ(*I*)] reflections	17455, 4084, 3881	43513, 4327, 4252	22839, 4542, 4276
*R*_int_	0.040	0.036	0.077
(sin θ/λ)_max_ (Å^−1^)	0.625	0.625	0.625

Refinement			
*R*[*F* ^2^ > 2σ(*F* ^2^)], *wR*(*F* ^2^), *S*	0.018, 0.040, 1.05	0.013, 0.033, 1.12	0.027, 0.068, 1.05
No. of reflections	4084	4327	4542
No. of parameters	247	257	275
No. of restraints	0	0	27
H-atom treatment	H-atom parameters constrained	H-atom parameters constrained	H-atom parameters constrained
Δρ_max_, Δρ_min_ (e Å^−3^)	0.53, −0.68	0.38, −0.92	1.87, −1.77

Computer programs: *CrysAlis PRO* (Rigaku OD, 2018 ▸), *SHELXS* (Sheldrick, 2008 ▸), *SHELXL* (Sheldrick, 2015 ▸), *SHELXL* 2016/4 (Sheldrick, 2015 ▸) and *OLEX2* (Dolomanov *et al.*, 2009 ▸).

## supplementary crystallographic information

### (η2-2-Allyl-4-methoxy-5-{[(propan-2-yloxy)carbonyl]methoxy}phenyl-κC1)chlorido(pyridine-κN)platinum(II) (I) Crystal data

**Table:**

[Pt(C_15_H_19_O_4_)Cl(C_5_H_5_N)]	*Z* = 2
*M**_r_* = 572.94	*F*(000) = 556
Triclinic, *P*1	*D*_x_ = 1.908 Mg m^−^^3^
*a* = 8.3146 (3) Å	Mo *K*α radiation, λ = 0.71073 Å
*b* = 8.6714 (4) Å	Cell parameters from 12348 reflections
*c* = 14.5827 (6) Å	θ = 3.2–29.0°
α = 90.534 (4)°	µ = 7.19 mm^−^^1^
β = 104.376 (4)°	*T* = 100 K
γ = 101.135 (3)°	Needle, clear colourless
*V* = 997.49 (7) Å^3^	0.25 × 0.2 × 0.15 mm

### (η2-2-Allyl-4-methoxy-5-{[(propan-2-yloxy)carbonyl]methoxy}phenyl-κC1)chlorido(pyridine-κN)platinum(II) (I) Data collection

**Table:**

Rigaku Oxford Diffraction SuperNova, Single source at offset/far, Eos diffractometer	4084 independent reflections
Radiation source: SuperNova (Mo) X-ray Source	3881 reflections with *I* > 2σ(*I*)
Mirror monochromator	*R*_int_ = 0.040
Detector resolution: 15.9631 pixels mm^-1^	θ_max_ = 26.4°, θ_min_ = 2.7°
ω scans	*h* = −10→10
Absorption correction: multi-scan CrysAlisPro (Rigaku OD, 2018)	*k* = −10→10
*T*_min_ = 0.717, *T*_max_ = 1.000	*l* = −18→18
17455 measured reflections	

### (η2-2-Allyl-4-methoxy-5-{[(propan-2-yloxy)carbonyl]methoxy}phenyl-κC1)chlorido(pyridine-κN)platinum(II) (I) Refinement

**Table:**

Refinement on *F*^2^	Primary atom site location: heavy-atom method
Least-squares matrix: full	Hydrogen site location: inferred from neighbouring sites
*R*[*F*^2^ > 2σ(*F*^2^)] = 0.018	H-atom parameters constrained
*wR*(*F*^2^) = 0.040	*w* = 1/[σ^2^(*F*_o_^2^) + (0.0133*P*)^2^ + 0.0785*P*] where *P* = (*F*_o_^2^ + 2*F*_c_^2^)/3
*S* = 1.05	(Δ/σ)_max_ = 0.001
4084 reflections	Δρ_max_ = 0.53 e Å^−^^3^
247 parameters	Δρ_min_ = −0.68 e Å^−^^3^
0 restraints	

### (η2-2-Allyl-4-methoxy-5-{[(propan-2-yloxy)carbonyl]methoxy}phenyl-κC1)chlorido(pyridine-κN)platinum(II) (I) Special details

**Table:**

Geometry. All esds (except the esd in the dihedral angle between two l.s. planes) are estimated using the full covariance matrix. The cell esds are taken into account individually in the estimation of esds in distances, angles and torsion angles; correlations between esds in cell parameters are only used when they are defined by crystal symmetry. An approximate (isotropic) treatment of cell esds is used for estimating esds involving l.s. planes.

### (η2-2-Allyl-4-methoxy-5-{[(propan-2-yloxy)carbonyl]methoxy}phenyl-κC1)chlorido(pyridine-κN)platinum(II) (I) Fractional atomic coordinates and isotropic or equivalent isotropic displacement parameters (Å^2^)

**Table:**

	*x*	*y*	*z*	*U*_iso_*/*U*_eq_	
Pt1	0.99983 (2)	0.56342 (2)	0.66724 (2)	0.00936 (4)	
C2	0.9123 (4)	0.3610 (3)	0.7197 (2)	0.0114 (6)	
C3	0.7406 (3)	0.2958 (3)	0.6843 (2)	0.0112 (6)	
C4	0.6695 (4)	0.1536 (3)	0.7156 (2)	0.0118 (6)	
H4	0.552907	0.109068	0.689752	0.014*	
C5	0.7671 (4)	0.0764 (3)	0.7842 (2)	0.0109 (6)	
C6	0.9385 (3)	0.1462 (3)	0.8242 (2)	0.0107 (6)	
C7	1.0104 (4)	0.2848 (3)	0.79078 (19)	0.0111 (6)	
H7	1.127197	0.328942	0.816169	0.013*	
C8	0.6398 (4)	0.3846 (3)	0.6098 (2)	0.0159 (7)	
H8A	0.527756	0.383967	0.622071	0.019*	
H8B	0.621058	0.332488	0.546305	0.019*	
C9	0.7352 (3)	0.5535 (3)	0.6122 (2)	0.0165 (7)	
H9	0.755404	0.595040	0.555064	0.020*	
C10	0.7937 (4)	0.6486 (4)	0.6956 (2)	0.0211 (7)	
H10A	0.774433	0.608533	0.753176	0.025*	
H10B	0.853053	0.753727	0.695070	0.025*	
O11	0.7105 (2)	−0.0634 (2)	0.81928 (13)	0.0131 (4)	
C12	0.5418 (3)	−0.1455 (3)	0.7741 (2)	0.0149 (7)	
H12A	0.530511	−0.162299	0.706052	0.022*	
H12B	0.460286	−0.082864	0.783549	0.022*	
H12C	0.519246	−0.247470	0.801796	0.022*	
O13	1.0221 (2)	0.0671 (2)	0.89596 (14)	0.0170 (5)	
C14	1.1676 (4)	0.1557 (4)	0.9612 (2)	0.0159 (7)	
H14A	1.180615	0.108580	1.023559	0.019*	
H14B	1.151072	0.264493	0.969509	0.019*	
C15	1.3272 (4)	0.1608 (3)	0.9288 (2)	0.0127 (6)	
O16	1.3354 (3)	0.0981 (2)	0.85702 (14)	0.0194 (5)	
O17	1.4603 (2)	0.2461 (2)	0.99284 (14)	0.0159 (5)	
C18	1.6258 (4)	0.2675 (4)	0.9695 (2)	0.0175 (7)	
H18	1.635179	0.168389	0.937456	0.021*	
C19	1.7574 (4)	0.2994 (4)	1.0648 (2)	0.0248 (8)	
H19A	1.743163	0.392815	1.097858	0.037*	
H19B	1.871372	0.317564	1.054367	0.037*	
H19C	1.742192	0.208415	1.103366	0.037*	
C20	1.6394 (4)	0.4002 (4)	0.9044 (2)	0.0204 (7)	
H20A	1.549167	0.373773	0.845589	0.031*	
H20B	1.749964	0.416659	0.889566	0.031*	
H20C	1.627884	0.496738	0.935450	0.031*	
Cl21	1.25878 (9)	0.49780 (8)	0.67051 (5)	0.01618 (16)	
N22	1.0890 (3)	0.7834 (3)	0.61344 (17)	0.0113 (5)	
C23	1.1235 (4)	0.9189 (3)	0.6672 (2)	0.0140 (6)	
H23	1.099025	0.916392	0.727521	0.017*	
C24	1.1935 (4)	1.0613 (3)	0.6375 (2)	0.0157 (7)	
H24	1.216748	1.155279	0.676646	0.019*	
C25	1.2290 (4)	1.0645 (4)	0.5497 (2)	0.0168 (7)	
H25	1.277421	1.160967	0.527790	0.020*	
C26	1.1935 (4)	0.9263 (4)	0.4941 (2)	0.0169 (7)	
H26	1.216671	0.926321	0.433546	0.020*	
C27	1.1239 (4)	0.7885 (3)	0.5282 (2)	0.0136 (6)	
H27	1.099668	0.693346	0.490065	0.016*	

### (η2-2-Allyl-4-methoxy-5-{[(propan-2-yloxy)carbonyl]methoxy}phenyl-κC1)chlorido(pyridine-κN)platinum(II) (I) Atomic displacement parameters (Å^2^)

**Table:**

	*U*^11^	*U*^22^	*U*^33^	*U*^12^	*U*^13^	*U*^23^
Pt1	0.00967 (6)	0.00807 (6)	0.01093 (7)	0.00194 (4)	0.00356 (5)	0.00123 (4)
C2	0.0130 (15)	0.0100 (14)	0.0118 (15)	0.0020 (11)	0.0044 (12)	−0.0017 (12)
C3	0.0122 (14)	0.0114 (14)	0.0104 (14)	0.0028 (11)	0.0035 (12)	−0.0017 (12)
C4	0.0105 (14)	0.0130 (15)	0.0113 (15)	0.0007 (11)	0.0031 (12)	−0.0014 (12)
C5	0.0160 (15)	0.0102 (14)	0.0092 (14)	0.0018 (11)	0.0088 (12)	0.0000 (12)
C6	0.0103 (14)	0.0106 (14)	0.0105 (14)	0.0038 (11)	0.0002 (12)	0.0004 (12)
C7	0.0092 (14)	0.0132 (15)	0.0110 (15)	0.0014 (11)	0.0036 (12)	0.0003 (12)
C8	0.0119 (15)	0.0150 (16)	0.0212 (17)	0.0039 (12)	0.0041 (13)	0.0050 (13)
C9	0.0084 (14)	0.0181 (16)	0.0261 (18)	0.0068 (12)	0.0070 (13)	0.0089 (14)
C10	0.0184 (17)	0.0163 (16)	0.034 (2)	0.0053 (13)	0.0153 (15)	0.0048 (15)
O11	0.0121 (10)	0.0111 (10)	0.0122 (10)	−0.0033 (8)	0.0003 (8)	0.0022 (8)
C12	0.0095 (14)	0.0149 (15)	0.0175 (16)	−0.0030 (12)	0.0021 (12)	0.0005 (13)
O13	0.0117 (11)	0.0160 (11)	0.0168 (11)	−0.0030 (8)	−0.0040 (9)	0.0072 (9)
C14	0.0125 (15)	0.0213 (17)	0.0104 (15)	−0.0008 (13)	−0.0003 (12)	0.0051 (13)
C15	0.0132 (15)	0.0096 (14)	0.0140 (15)	0.0023 (11)	0.0012 (12)	0.0037 (12)
O16	0.0184 (12)	0.0210 (12)	0.0160 (11)	0.0036 (9)	−0.0004 (9)	−0.0072 (10)
O17	0.0106 (10)	0.0203 (11)	0.0137 (11)	−0.0026 (8)	0.0019 (9)	−0.0044 (9)
C18	0.0124 (15)	0.0224 (17)	0.0183 (16)	0.0009 (13)	0.0073 (13)	−0.0015 (14)
C19	0.0159 (17)	0.032 (2)	0.0223 (18)	−0.0008 (14)	0.0018 (14)	0.0025 (16)
C20	0.0222 (17)	0.0204 (17)	0.0188 (17)	−0.0016 (13)	0.0100 (14)	−0.0001 (14)
Cl21	0.0109 (3)	0.0124 (4)	0.0265 (4)	0.0028 (3)	0.0067 (3)	0.0034 (3)
N22	0.0079 (12)	0.0111 (12)	0.0140 (13)	0.0013 (9)	0.0013 (10)	0.0014 (10)
C23	0.0136 (15)	0.0151 (15)	0.0126 (15)	0.0052 (12)	0.0001 (12)	0.0017 (13)
C24	0.0147 (15)	0.0122 (15)	0.0189 (16)	0.0020 (12)	0.0027 (13)	−0.0023 (13)
C25	0.0129 (15)	0.0138 (15)	0.0215 (17)	0.0011 (12)	0.0011 (13)	0.0056 (13)
C26	0.0190 (16)	0.0212 (17)	0.0131 (15)	0.0057 (13)	0.0075 (13)	0.0044 (13)
C27	0.0121 (15)	0.0149 (15)	0.0142 (15)	0.0046 (12)	0.0024 (12)	0.0012 (13)

### (η2-2-Allyl-4-methoxy-5-{[(propan-2-yloxy)carbonyl]methoxy}phenyl-κC1)chlorido(pyridine-κN)platinum(II) (I) Geometric parameters (Å, º)

**Table:**

Pt1—C2	2.001 (3)	O13—C14	1.419 (3)
Pt1—C9	2.131 (3)	C14—H14A	0.9900
Pt1—C10	2.118 (3)	C14—H14B	0.9900
Pt1—Cl21	2.3205 (7)	C14—C15	1.509 (4)
Pt1—N22	2.139 (2)	C15—O16	1.198 (3)
C2—C3	1.393 (4)	C15—O17	1.342 (3)
C2—C7	1.407 (4)	O17—C18	1.476 (3)
C3—C4	1.393 (4)	C18—H18	1.0000
C3—C8	1.513 (4)	C18—C19	1.525 (4)
C4—H4	0.9500	C18—C20	1.505 (4)
C4—C5	1.388 (4)	C19—H19A	0.9800
C5—C6	1.409 (4)	C19—H19B	0.9800
C5—O11	1.365 (3)	C19—H19C	0.9800
C6—C7	1.384 (4)	C20—H20A	0.9800
C6—O13	1.373 (3)	C20—H20B	0.9800
C7—H7	0.9500	C20—H20C	0.9800
C8—H8A	0.9900	N22—C23	1.349 (4)
C8—H8B	0.9900	N22—C27	1.344 (4)
C8—C9	1.520 (4)	C23—H23	0.9500
C9—H9	0.9500	C23—C24	1.381 (4)
C9—C10	1.389 (4)	C24—H24	0.9500
C10—H10A	0.9500	C24—C25	1.383 (4)
C10—H10B	0.9500	C25—H25	0.9500
O11—C12	1.434 (3)	C25—C26	1.380 (4)
C12—H12A	0.9800	C26—H26	0.9500
C12—H12B	0.9800	C26—C27	1.378 (4)
C12—H12C	0.9800	C27—H27	0.9500
			
C2—Pt1—C9	81.36 (12)	H12A—C12—H12B	109.5
C2—Pt1—C10	87.41 (12)	H12A—C12—H12C	109.5
C2—Pt1—Cl21	93.43 (9)	H12B—C12—H12C	109.5
C2—Pt1—N22	178.21 (10)	C6—O13—C14	117.1 (2)
C9—Pt1—Cl21	154.99 (9)	O13—C14—H14A	109.1
C9—Pt1—N22	97.67 (10)	O13—C14—H14B	109.1
C10—Pt1—C9	38.16 (12)	O13—C14—C15	112.3 (2)
C10—Pt1—Cl21	166.72 (9)	H14A—C14—H14B	107.9
C10—Pt1—N22	90.90 (11)	C15—C14—H14A	109.1
N22—Pt1—Cl21	88.08 (7)	C15—C14—H14B	109.1
C3—C2—Pt1	116.6 (2)	O16—C15—C14	125.5 (3)
C3—C2—C7	118.8 (3)	O16—C15—O17	124.6 (3)
C7—C2—Pt1	124.6 (2)	O17—C15—C14	109.8 (2)
C2—C3—C8	116.7 (3)	C15—O17—C18	116.4 (2)
C4—C3—C2	120.5 (3)	O17—C18—H18	109.6
C4—C3—C8	122.8 (2)	O17—C18—C19	105.1 (2)
C3—C4—H4	119.6	O17—C18—C20	109.0 (3)
C5—C4—C3	120.7 (3)	C19—C18—H18	109.6
C5—C4—H4	119.6	C20—C18—H18	109.6
C4—C5—C6	119.1 (3)	C20—C18—C19	113.7 (3)
O11—C5—C4	125.5 (3)	C18—C19—H19A	109.5
O11—C5—C6	115.5 (3)	C18—C19—H19B	109.5
C7—C6—C5	120.0 (3)	C18—C19—H19C	109.5
O13—C6—C5	115.1 (2)	H19A—C19—H19B	109.5
O13—C6—C7	124.9 (2)	H19A—C19—H19C	109.5
C2—C7—H7	119.6	H19B—C19—H19C	109.5
C6—C7—C2	120.7 (3)	C18—C20—H20A	109.5
C6—C7—H7	119.6	C18—C20—H20B	109.5
C3—C8—H8A	109.7	C18—C20—H20C	109.5
C3—C8—H8B	109.7	H20A—C20—H20B	109.5
C3—C8—C9	109.8 (2)	H20A—C20—H20C	109.5
H8A—C8—H8B	108.2	H20B—C20—H20C	109.5
C9—C8—H8A	109.7	C23—N22—Pt1	120.7 (2)
C9—C8—H8B	109.7	C27—N22—Pt1	120.71 (19)
Pt1—C9—H9	90.2	C27—N22—C23	118.4 (2)
C8—C9—Pt1	109.4 (2)	N22—C23—H23	118.9
C8—C9—H9	119.1	N22—C23—C24	122.1 (3)
C10—C9—Pt1	70.44 (17)	C24—C23—H23	118.9
C10—C9—C8	121.7 (3)	C23—C24—H24	120.6
C10—C9—H9	119.1	C23—C24—C25	118.7 (3)
Pt1—C10—H10A	108.6	C25—C24—H24	120.6
Pt1—C10—H10B	90.0	C24—C25—H25	120.3
C9—C10—Pt1	71.40 (18)	C26—C25—C24	119.4 (3)
C9—C10—H10A	120.0	C26—C25—H25	120.3
C9—C10—H10B	120.0	C25—C26—H26	120.6
H10A—C10—H10B	120.0	C27—C26—C25	118.8 (3)
C5—O11—C12	117.1 (2)	C27—C26—H26	120.6
O11—C12—H12A	109.5	N22—C27—C26	122.5 (3)
O11—C12—H12B	109.5	N22—C27—H27	118.7
O11—C12—H12C	109.5	C26—C27—H27	118.7
			
Pt1—C2—C3—C4	178.9 (2)	C7—C2—C3—C4	−3.1 (4)
Pt1—C2—C3—C8	0.3 (3)	C7—C2—C3—C8	178.4 (3)
Pt1—C2—C7—C6	178.8 (2)	C7—C6—O13—C14	22.9 (4)
Pt1—N22—C23—C24	174.9 (2)	C8—C3—C4—C5	−179.9 (3)
Pt1—N22—C27—C26	−175.0 (2)	C8—C9—C10—Pt1	101.2 (3)
C2—C3—C4—C5	1.6 (4)	O11—C5—C6—C7	177.3 (2)
C2—C3—C8—C9	−18.2 (4)	O11—C5—C6—O13	−2.8 (4)
C3—C2—C7—C6	0.9 (4)	O13—C6—C7—C2	−177.2 (3)
C3—C4—C5—C6	2.1 (4)	O13—C14—C15—O16	−0.4 (4)
C3—C4—C5—O11	−179.6 (3)	O13—C14—C15—O17	179.9 (2)
C3—C8—C9—Pt1	25.9 (3)	C14—C15—O17—C18	−177.7 (2)
C3—C8—C9—C10	−52.5 (4)	C15—O17—C18—C19	−155.7 (2)
C4—C3—C8—C9	163.3 (3)	C15—O17—C18—C20	82.1 (3)
C4—C5—C6—C7	−4.2 (4)	O16—C15—O17—C18	2.6 (4)
C4—C5—C6—O13	175.7 (3)	N22—C23—C24—C25	0.0 (4)
C4—C5—O11—C12	7.4 (4)	C23—N22—C27—C26	0.1 (4)
C5—C6—C7—C2	2.7 (4)	C23—C24—C25—C26	0.2 (4)
C5—C6—O13—C14	−156.9 (3)	C24—C25—C26—C27	−0.2 (4)
C6—C5—O11—C12	−174.1 (2)	C25—C26—C27—N22	0.1 (5)
C6—O13—C14—C15	−88.0 (3)	C27—N22—C23—C24	−0.1 (4)

### (η2-2-Allyl-4-methoxy-5-{[(propan-2-yloxy)carbonyl]methoxy}phenyl-κC1)chlorido(pyridine-κN)platinum(II) (I) Hydrogen-bond geometry (Å, º)

*Cg*5 is the centroid of the C2–C7 phenyl ring.

**Table:**

*D*—H···*A*	*D*—H	H···*A*	*D*···*A*	*D*—H···*A*
C12—H12*B*···O16^i^	0.98	2.42	3.354 (3)	160
C14—H14*A*···O11^ii^	0.99	2.31	3.266 (3)	161
C14—H14*A*···O13^ii^	0.99	2.56	3.330 (4)	134
C20—H20*B*···*Cg*5^iii^	0.98	2.93	3.586 (3)	125
C26—H26···*Cg*5^iv^	0.95	2.88	3.736 (3)	150

Symmetry codes: (i) *x*−1, *y*, *z*; (ii) −*x*+2, −*y*, −*z*+2; (iii) *x*+1, *y*, *z*; (iv) −*x*+2, −*y*+1, −*z*+1.

### (η2-2-Allyl-4-methoxy-5-{[(propan-2-yloxy)carbonyl]methoxy}phenyl-κC1)chlorido(4-methylpyridine-κN)platinum(II) (II) Crystal data

**Table:**

[Pt(C_15_H_19_O_4_)Cl(C_6_H_7_N)]	*Z* = 2
*M**_r_* = 586.97	*F*(000) = 572
Triclinic, *P*1	*D*_x_ = 1.841 Mg m^−^^3^
*a* = 8.36089 (15) Å	Mo *K*α radiation, λ = 0.71073 Å
*b* = 9.12717 (16) Å	Cell parameters from 35745 reflections
*c* = 14.5582 (3) Å	θ = 3.2–29.0°
α = 94.9089 (15)°	µ = 6.78 mm^−^^1^
β = 102.2766 (16)°	*T* = 100 K
γ = 100.4541 (15)°	Needle, colourless
*V* = 1058.58 (3) Å^3^	0.25 × 0.2 × 0.2 mm

### (η2-2-Allyl-4-methoxy-5-{[(propan-2-yloxy)carbonyl]methoxy}phenyl-κC1)chlorido(4-methylpyridine-κN)platinum(II) (II) Data collection

**Table:**

Rigaku Oxford Diffraction SuperNova, Single source at offset/far, Eos diffractometer	4327 independent reflections
Radiation source: SuperNova (Mo) X-ray Source	4252 reflections with *I* > 2σ(*I*)
Mirror monochromator	*R*_int_ = 0.036
Detector resolution: 15.9631 pixels mm^-1^	θ_max_ = 26.4°, θ_min_ = 2.9°
ω scans	*h* = −10→10
Absorption correction: multi-scan CrysAlisPro (Rigaku OD, 2018)	*k* = −11→11
*T*_min_ = 0.671, *T*_max_ = 1.000	*l* = −18→18
43513 measured reflections	

### (η2-2-Allyl-4-methoxy-5-{[(propan-2-yloxy)carbonyl]methoxy}phenyl-κC1)chlorido(4-methylpyridine-κN)platinum(II) (II) Refinement

**Table:**

Refinement on *F*^2^	Primary atom site location: structure-invariant direct methods
Least-squares matrix: full	Hydrogen site location: inferred from neighbouring sites
*R*[*F*^2^ > 2σ(*F*^2^)] = 0.013	H-atom parameters constrained
*wR*(*F*^2^) = 0.033	*w* = 1/[σ^2^(*F*_o_^2^) + (0.015*P*)^2^ + 0.659*P*] where *P* = (*F*_o_^2^ + 2*F*_c_^2^)/3
*S* = 1.12	(Δ/σ)_max_ = 0.005
4327 reflections	Δρ_max_ = 0.38 e Å^−^^3^
257 parameters	Δρ_min_ = −0.92 e Å^−^^3^
0 restraints	

### (η2-2-Allyl-4-methoxy-5-{[(propan-2-yloxy)carbonyl]methoxy}phenyl-κC1)chlorido(4-methylpyridine-κN)platinum(II) (II) Special details

**Table:**

Geometry. All esds (except the esd in the dihedral angle between two l.s. planes) are estimated using the full covariance matrix. The cell esds are taken into account individually in the estimation of esds in distances, angles and torsion angles; correlations between esds in cell parameters are only used when they are defined by crystal symmetry. An approximate (isotropic) treatment of cell esds is used for estimating esds involving l.s. planes.

### (η2-2-Allyl-4-methoxy-5-{[(propan-2-yloxy)carbonyl]methoxy}phenyl-κC1)chlorido(4-methylpyridine-κN)platinum(II) (II) Fractional atomic coordinates and isotropic or equivalent isotropic displacement parameters (Å^2^)

**Table:**

	*x*	*y*	*z*	*U*_iso_*/*U*_eq_	
Pt1	1.00923 (2)	0.54971 (2)	0.69268 (2)	0.00989 (3)	
C2	0.9207 (3)	0.3594 (2)	0.74088 (15)	0.0119 (4)	
C3	0.7507 (3)	0.2995 (2)	0.70567 (15)	0.0126 (4)	
C4	0.6771 (3)	0.1616 (2)	0.72883 (15)	0.0122 (4)	
H4	0.562025	0.120185	0.702254	0.015*	
C5	0.7718 (3)	0.0851 (2)	0.79059 (15)	0.0119 (4)	
C6	0.9409 (3)	0.1511 (2)	0.83258 (15)	0.0124 (4)	
C7	1.0146 (3)	0.2846 (2)	0.80607 (15)	0.0126 (4)	
H7	1.129836	0.325802	0.832241	0.015*	
C8	0.6526 (3)	0.3880 (2)	0.63896 (16)	0.0163 (4)	
H8A	0.632773	0.340077	0.572804	0.020*	
H8B	0.542682	0.387817	0.654178	0.020*	
C9	0.7486 (3)	0.5483 (2)	0.64848 (17)	0.0176 (5)	
H9	0.764409	0.589874	0.592650	0.021*	
C10	0.8151 (3)	0.6376 (3)	0.73626 (18)	0.0202 (5)	
H10A	0.800671	0.598144	0.792944	0.024*	
H10B	0.874500	0.737636	0.739110	0.024*	
O11	0.71319 (18)	−0.05067 (16)	0.81844 (11)	0.0144 (3)	
C12	0.5483 (3)	−0.1295 (2)	0.76921 (16)	0.0164 (4)	
H12A	0.542265	−0.144001	0.700981	0.025*	
H12B	0.466172	−0.070730	0.781434	0.025*	
H12C	0.524050	−0.227516	0.791625	0.025*	
O13	1.02076 (19)	0.07216 (17)	0.89891 (11)	0.0171 (3)	
C14	1.1632 (3)	0.1545 (2)	0.96761 (15)	0.0155 (4)	
H14A	1.173924	0.106448	1.026448	0.019*	
H14B	1.147068	0.257814	0.982931	0.019*	
C15	1.3235 (3)	0.1620 (2)	0.93298 (15)	0.0134 (4)	
O16	1.3310 (2)	0.10801 (18)	0.85616 (11)	0.0201 (3)	
O17	1.45437 (18)	0.23658 (17)	1.00086 (10)	0.0160 (3)	
C18	1.6184 (3)	0.2570 (3)	0.97671 (16)	0.0165 (4)	
H18	1.623467	0.166368	0.934555	0.020*	
C19	1.7466 (3)	0.2720 (3)	1.06927 (18)	0.0277 (6)	
H19A	1.858792	0.285300	1.056968	0.042*	
H19B	1.724572	0.181059	1.099656	0.042*	
H19C	1.739589	0.359312	1.111216	0.042*	
C20	1.6403 (3)	0.3939 (3)	0.92522 (18)	0.0239 (5)	
H20A	1.637086	0.483010	0.966895	0.036*	
H20B	1.549879	0.379957	0.868030	0.036*	
H20C	1.748194	0.407443	0.907410	0.036*	
Cl21	1.26067 (6)	0.48086 (6)	0.68589 (4)	0.01889 (11)	
N22	1.0906 (2)	0.74891 (19)	0.63327 (13)	0.0129 (4)	
C23	1.1236 (3)	0.8889 (2)	0.68027 (15)	0.0135 (4)	
H23	1.107292	0.901003	0.742873	0.016*	
C24	1.1802 (3)	1.0157 (2)	0.64104 (15)	0.0147 (4)	
H24	1.203176	1.112415	0.676689	0.018*	
C25	1.2034 (3)	1.0004 (2)	0.54849 (16)	0.0146 (4)	
C26	1.1654 (3)	0.8558 (2)	0.49975 (16)	0.0175 (5)	
H26	1.176376	0.840720	0.436193	0.021*	
C27	1.1117 (3)	0.7344 (2)	0.54383 (16)	0.0168 (4)	
H27	1.088589	0.636514	0.509765	0.020*	
C28	1.2680 (3)	1.1334 (3)	0.50278 (17)	0.0195 (5)	
H28A	1.256524	1.225818	0.538017	0.029*	
H28B	1.203488	1.123624	0.437162	0.029*	
H28C	1.386158	1.137376	0.503370	0.029*	

### (η2-2-Allyl-4-methoxy-5-{[(propan-2-yloxy)carbonyl]methoxy}phenyl-κC1)chlorido(4-methylpyridine-κN)platinum(II) (II) Atomic displacement parameters (Å^2^)

**Table:**

	*U*^11^	*U*^22^	*U*^33^	*U*^12^	*U*^13^	*U*^23^
Pt1	0.00854 (5)	0.00956 (5)	0.01200 (5)	0.00219 (3)	0.00279 (3)	0.00204 (3)
C2	0.0118 (10)	0.0109 (10)	0.0139 (10)	0.0034 (8)	0.0048 (8)	0.0004 (8)
C3	0.0119 (10)	0.0141 (10)	0.0136 (10)	0.0058 (8)	0.0038 (8)	0.0023 (8)
C4	0.0073 (9)	0.0153 (10)	0.0139 (10)	0.0024 (8)	0.0027 (8)	0.0005 (8)
C5	0.0113 (10)	0.0112 (10)	0.0140 (10)	0.0011 (8)	0.0055 (8)	0.0014 (8)
C6	0.0111 (10)	0.0137 (10)	0.0127 (10)	0.0044 (8)	0.0011 (8)	0.0026 (8)
C7	0.0090 (10)	0.0145 (10)	0.0133 (10)	0.0016 (8)	0.0013 (8)	0.0014 (8)
C8	0.0108 (10)	0.0175 (11)	0.0209 (12)	0.0042 (8)	0.0019 (9)	0.0060 (9)
C9	0.0095 (10)	0.0191 (11)	0.0274 (13)	0.0063 (9)	0.0058 (9)	0.0098 (9)
C10	0.0173 (11)	0.0149 (11)	0.0339 (14)	0.0066 (9)	0.0148 (10)	0.0045 (10)
O11	0.0092 (7)	0.0125 (7)	0.0189 (8)	−0.0015 (6)	−0.0005 (6)	0.0044 (6)
C12	0.0090 (10)	0.0145 (10)	0.0229 (12)	−0.0014 (8)	0.0012 (9)	0.0016 (9)
O13	0.0109 (7)	0.0157 (7)	0.0203 (8)	−0.0019 (6)	−0.0045 (6)	0.0080 (6)
C14	0.0112 (10)	0.0185 (11)	0.0138 (11)	−0.0009 (8)	−0.0010 (9)	0.0043 (9)
C15	0.0119 (10)	0.0112 (10)	0.0148 (11)	0.0015 (8)	−0.0020 (8)	0.0032 (8)
O16	0.0172 (8)	0.0239 (8)	0.0160 (8)	0.0047 (7)	−0.0004 (7)	−0.0047 (7)
O17	0.0092 (7)	0.0218 (8)	0.0136 (8)	−0.0023 (6)	0.0020 (6)	−0.0021 (6)
C18	0.0105 (10)	0.0200 (11)	0.0179 (11)	−0.0001 (8)	0.0051 (9)	−0.0017 (9)
C19	0.0129 (11)	0.0416 (15)	0.0245 (13)	−0.0005 (11)	0.0011 (10)	0.0027 (11)
C20	0.0212 (12)	0.0226 (12)	0.0291 (14)	0.0001 (10)	0.0123 (11)	0.0039 (10)
Cl21	0.0115 (2)	0.0175 (3)	0.0308 (3)	0.0050 (2)	0.0091 (2)	0.0056 (2)
N22	0.0119 (9)	0.0119 (8)	0.0145 (9)	0.0022 (7)	0.0020 (7)	0.0028 (7)
C23	0.0118 (10)	0.0159 (10)	0.0108 (10)	0.0021 (8)	−0.0007 (8)	0.0007 (8)
C24	0.0127 (10)	0.0142 (10)	0.0140 (11)	0.0018 (8)	−0.0023 (8)	−0.0002 (8)
C25	0.0088 (10)	0.0170 (11)	0.0171 (11)	0.0021 (8)	0.0001 (8)	0.0046 (9)
C26	0.0199 (11)	0.0189 (11)	0.0153 (11)	0.0044 (9)	0.0073 (9)	0.0025 (9)
C27	0.0194 (11)	0.0143 (10)	0.0163 (11)	0.0021 (9)	0.0059 (9)	−0.0021 (8)
C28	0.0195 (12)	0.0180 (11)	0.0195 (12)	0.0008 (9)	0.0029 (10)	0.0059 (9)

### (η2-2-Allyl-4-methoxy-5-{[(propan-2-yloxy)carbonyl]methoxy}phenyl-κC1)chlorido(4-methylpyridine-κN)platinum(II) (II) Geometric parameters (Å, º)

**Table:**

Pt1—C2	2.005 (2)	C14—H14B	0.9900
Pt1—C9	2.134 (2)	C14—C15	1.521 (3)
Pt1—C10	2.123 (2)	C15—O16	1.202 (3)
Pt1—Cl21	2.3197 (5)	C15—O17	1.339 (3)
Pt1—N22	2.1418 (18)	O17—C18	1.471 (3)
C2—C3	1.393 (3)	C18—H18	1.0000
C2—C7	1.405 (3)	C18—C19	1.512 (3)
C3—C4	1.399 (3)	C18—C20	1.514 (3)
C3—C8	1.518 (3)	C19—H19A	0.9800
C4—H4	0.9500	C19—H19B	0.9800
C4—C5	1.388 (3)	C19—H19C	0.9800
C5—C6	1.413 (3)	C20—H20A	0.9800
C5—O11	1.375 (2)	C20—H20B	0.9800
C6—C7	1.387 (3)	C20—H20C	0.9800
C6—O13	1.384 (2)	N22—C23	1.348 (3)
C7—H7	0.9500	N22—C27	1.349 (3)
C8—H8A	0.9900	C23—H23	0.9500
C8—H8B	0.9900	C23—C24	1.386 (3)
C8—C9	1.515 (3)	C24—H24	0.9500
C9—H9	0.9500	C24—C25	1.400 (3)
C9—C10	1.401 (3)	C25—C26	1.392 (3)
C10—H10A	0.9500	C25—C28	1.503 (3)
C10—H10B	0.9500	C26—H26	0.9500
O11—C12	1.436 (2)	C26—C27	1.380 (3)
C12—H12A	0.9800	C27—H27	0.9500
C12—H12B	0.9800	C28—H28A	0.9800
C12—H12C	0.9800	C28—H28B	0.9800
O13—C14	1.423 (3)	C28—H28C	0.9800
C14—H14A	0.9900		
			
C2—Pt1—C9	81.60 (8)	C6—O13—C14	117.11 (16)
C2—Pt1—C10	86.78 (9)	O13—C14—H14A	109.2
C2—Pt1—Cl21	93.24 (6)	O13—C14—H14B	109.2
C2—Pt1—N22	176.29 (7)	O13—C14—C15	112.13 (18)
C9—Pt1—Cl21	156.66 (7)	H14A—C14—H14B	107.9
C9—Pt1—N22	95.50 (8)	C15—C14—H14A	109.2
C10—Pt1—C9	38.42 (9)	C15—C14—H14B	109.2
C10—Pt1—Cl21	164.63 (7)	O16—C15—C14	124.81 (19)
C10—Pt1—N22	92.37 (8)	O16—C15—O17	125.2 (2)
N22—Pt1—Cl21	88.53 (5)	O17—C15—C14	109.96 (18)
C3—C2—Pt1	115.65 (15)	C15—O17—C18	116.30 (16)
C3—C2—C7	118.71 (19)	O17—C18—H18	109.5
C7—C2—Pt1	125.64 (16)	O17—C18—C19	106.23 (18)
C2—C3—C4	120.89 (19)	O17—C18—C20	108.67 (18)
C2—C3—C8	116.84 (19)	C19—C18—H18	109.5
C4—C3—C8	122.23 (19)	C19—C18—C20	113.4 (2)
C3—C4—H4	119.9	C20—C18—H18	109.5
C5—C4—C3	120.14 (19)	C18—C19—H19A	109.5
C5—C4—H4	119.9	C18—C19—H19B	109.5
C4—C5—C6	119.24 (19)	C18—C19—H19C	109.5
O11—C5—C4	125.30 (19)	H19A—C19—H19B	109.5
O11—C5—C6	115.42 (18)	H19A—C19—H19C	109.5
C7—C6—C5	120.07 (19)	H19B—C19—H19C	109.5
O13—C6—C5	114.89 (18)	C18—C20—H20A	109.5
O13—C6—C7	125.04 (19)	C18—C20—H20B	109.5
C2—C7—H7	119.7	C18—C20—H20C	109.5
C6—C7—C2	120.66 (19)	H20A—C20—H20B	109.5
C6—C7—H7	119.7	H20A—C20—H20C	109.5
C3—C8—H8A	109.6	H20B—C20—H20C	109.5
C3—C8—H8B	109.6	C23—N22—Pt1	123.87 (15)
H8A—C8—H8B	108.2	C23—N22—C27	117.67 (18)
C9—C8—C3	110.11 (18)	C27—N22—Pt1	118.46 (14)
C9—C8—H8A	109.6	N22—C23—H23	118.6
C9—C8—H8B	109.6	N22—C23—C24	122.7 (2)
Pt1—C9—H9	91.2	C24—C23—H23	118.6
C8—C9—Pt1	108.44 (14)	C23—C24—H24	120.2
C8—C9—H9	118.7	C23—C24—C25	119.5 (2)
C10—C9—Pt1	70.35 (12)	C25—C24—H24	120.2
C10—C9—C8	122.7 (2)	C24—C25—C28	122.0 (2)
C10—C9—H9	118.7	C26—C25—C24	117.3 (2)
Pt1—C10—H10A	107.5	C26—C25—C28	120.6 (2)
Pt1—C10—H10B	91.2	C25—C26—H26	120.0
C9—C10—Pt1	71.23 (12)	C27—C26—C25	119.9 (2)
C9—C10—H10A	120.0	C27—C26—H26	120.0
C9—C10—H10B	120.0	N22—C27—C26	122.8 (2)
H10A—C10—H10B	120.0	N22—C27—H27	118.6
C5—O11—C12	116.96 (16)	C26—C27—H27	118.6
O11—C12—H12A	109.5	C25—C28—H28A	109.5
O11—C12—H12B	109.5	C25—C28—H28B	109.5
O11—C12—H12C	109.5	C25—C28—H28C	109.5
H12A—C12—H12B	109.5	H28A—C28—H28B	109.5
H12A—C12—H12C	109.5	H28A—C28—H28C	109.5
H12B—C12—H12C	109.5	H28B—C28—H28C	109.5
			
Pt1—C2—C3—C4	175.43 (16)	C7—C2—C3—C8	177.26 (19)
Pt1—C2—C3—C8	−2.5 (2)	C7—C6—O13—C14	23.4 (3)
Pt1—C2—C7—C6	−178.28 (16)	C8—C3—C4—C5	−179.68 (19)
Pt1—N22—C23—C24	178.30 (15)	C8—C9—C10—Pt1	99.79 (19)
Pt1—N22—C27—C26	−179.30 (17)	O11—C5—C6—C7	176.98 (18)
C2—C3—C4—C5	2.5 (3)	O11—C5—C6—O13	−2.7 (3)
C2—C3—C8—C9	−17.9 (3)	O13—C6—C7—C2	−177.25 (19)
C3—C2—C7—C6	2.0 (3)	O13—C14—C15—O16	1.4 (3)
C3—C4—C5—C6	2.6 (3)	O13—C14—C15—O17	−178.19 (16)
C3—C4—C5—O11	179.99 (19)	C14—C15—O17—C18	−178.25 (17)
C3—C8—C9—Pt1	27.8 (2)	C15—O17—C18—C19	−153.51 (19)
C3—C8—C9—C10	−50.2 (3)	C15—O17—C18—C20	84.2 (2)
C4—C3—C8—C9	164.2 (2)	O16—C15—O17—C18	2.2 (3)
C4—C5—C6—C7	−5.4 (3)	N22—C23—C24—C25	0.7 (3)
C4—C5—C6—O13	174.91 (18)	C23—N22—C27—C26	0.2 (3)
C4—C5—O11—C12	9.6 (3)	C23—C24—C25—C26	0.8 (3)
C5—C6—C7—C2	3.1 (3)	C23—C24—C25—C28	−178.7 (2)
C5—C6—O13—C14	−156.95 (19)	C24—C25—C26—C27	−1.7 (3)
C6—C5—O11—C12	−172.89 (18)	C25—C26—C27—N22	1.3 (3)
C6—O13—C14—C15	−87.5 (2)	C27—N22—C23—C24	−1.2 (3)
C7—C2—C3—C4	−4.8 (3)	C28—C25—C26—C27	177.8 (2)

### (η2-2-Allyl-4-methoxy-5-{[(propan-2-yloxy)carbonyl]methoxy}phenyl-κC1)chlorido(4-methylpyridine-κN)platinum(II) (II) Hydrogen-bond geometry (Å, º)

*Cg*5 is the centroid of the C2–C7 phenyl ring.

**Table:**

*D*—H···*A*	*D*—H	H···*A*	*D*···*A*	*D*—H···*A*
C12—H12*B*···O16^i^	0.98	2.45	3.397 (3)	162
C14—H14*A*···O11^ii^	0.99	2.39	3.341 (3)	161
C14—H14*A*···O13^ii^	0.99	2.57	3.351 (3)	136
C8—H8*B*···Cl21^i^	0.99	2.76	3.713 (3)	162
C20—H20*B*···*Cg*5^iii^	0.98	2.87	3.562 (3)	128
C26—H26···*Cg*5^iv^	0.95	2.93	3.873 (3)	171
C28—H28*B*···*Cg*4^v^	0.98	2.87	3.425 (3)	117

Symmetry codes: (i) *x*−1, *y*, *z*; (ii) −*x*+2, −*y*, −*z*+2; (iii) *x*+1, *y*, *z*; (iv) −*x*+2, −*y*+1, −*z*+1; (v) −*x*+2, −*y*+2, −*z*+1.

### (η2-2-Allyl-4-methoxy-5-{[(propan-2-yloxy)carbonyl]methoxy}phenyl-κC1)chlorido(pyridine-4-carboxylic acid-κN)platinum(II) (III) Crystal data

**Table:**

[Pt(C_15_H_19_O_4_)Cl(C_6_H_5_NO_2_)]	*Z* = 2
*M**_r_* = 616.95	*F*(000) = 600
Triclinic, *P*1	*D*_x_ = 1.841 Mg m^−^^3^
*a* = 7.8746 (2) Å	Mo *K*α radiation, λ = 0.71073 Å
*b* = 9.7566 (2) Å	Cell parameters from 13370 reflections
*c* = 15.0004 (4) Å	θ = 2.8–29.1°
α = 95.782 (2)°	µ = 6.46 mm^−^^1^
β = 102.874 (2)°	*T* = 100 K
γ = 93.843 (2)°	Block, light yellow
*V* = 1113.02 (5) Å^3^	0.4 × 0.4 × 0.35 mm

### (η2-2-Allyl-4-methoxy-5-{[(propan-2-yloxy)carbonyl]methoxy}phenyl-κC1)chlorido(pyridine-4-carboxylic acid-κN)platinum(II) (III) Data collection

**Table:**

Rigaku Oxford Diffraction SuperNova, Single source at offset/far, Eos diffractometer	4542 independent reflections
Radiation source: micro-focus sealed X-ray tube, SuperNova (Mo) X-ray Source	4276 reflections with *I* > 2σ(*I*)
Mirror monochromator	*R*_int_ = 0.077
Detector resolution: 15.9631 pixels mm^-1^	θ_max_ = 26.4°, θ_min_ = 2.7°
ω scans	*h* = −9→9
Absorption correction: multi-scan CrysAlisPro (Rigaku OD, 2018)	*k* = −12→12
*T*_min_ = 0.429, *T*_max_ = 1.000	*l* = −18→18
22839 measured reflections	

### (η2-2-Allyl-4-methoxy-5-{[(propan-2-yloxy)carbonyl]methoxy}phenyl-κC1)chlorido(pyridine-4-carboxylic acid-κN)platinum(II) (III) Refinement

**Table:**

Refinement on *F*^2^	Primary atom site location: structure-invariant direct methods
Least-squares matrix: full	Hydrogen site location: inferred from neighbouring sites
*R*[*F*^2^ > 2σ(*F*^2^)] = 0.027	H-atom parameters constrained
*wR*(*F*^2^) = 0.068	*w* = 1/[σ^2^(*F*_o_^2^) + (0.0316*P*)^2^ + 0.7413*P*] where *P* = (*F*_o_^2^ + 2*F*_c_^2^)/3
*S* = 1.05	(Δ/σ)_max_ = 0.002
4542 reflections	Δρ_max_ = 1.87 e Å^−^^3^
275 parameters	Δρ_min_ = −1.77 e Å^−^^3^
27 restraints	

### (η2-2-Allyl-4-methoxy-5-{[(propan-2-yloxy)carbonyl]methoxy}phenyl-κC1)chlorido(pyridine-4-carboxylic acid-κN)platinum(II) (III) Special details

**Table:**

Geometry. All esds (except the esd in the dihedral angle between two l.s. planes) are estimated using the full covariance matrix. The cell esds are taken into account individually in the estimation of esds in distances, angles and torsion angles; correlations between esds in cell parameters are only used when they are defined by crystal symmetry. An approximate (isotropic) treatment of cell esds is used for estimating esds involving l.s. planes.

### (η2-2-Allyl-4-methoxy-5-{[(propan-2-yloxy)carbonyl]methoxy}phenyl-κC1)chlorido(pyridine-4-carboxylic acid-κN)platinum(II) (III) Fractional atomic coordinates and isotropic or equivalent isotropic displacement parameters (Å^2^)

**Table:**

	*x*	*y*	*z*	*U*_iso_*/*U*_eq_	
Pt1	−0.08260 (2)	0.65313 (2)	0.08249 (2)	0.01501 (7)	
C2	0.0203 (5)	0.6097 (4)	0.2108 (3)	0.0169 (8)	
C3	−0.0464 (5)	0.4856 (4)	0.2343 (3)	0.0187 (9)	
C4	0.0185 (5)	0.4418 (4)	0.3205 (3)	0.0183 (8)	
H4	−0.028452	0.357164	0.335382	0.022*	
C5	0.1523 (5)	0.5231 (4)	0.3843 (3)	0.0178 (9)	
C6	0.2201 (5)	0.6479 (4)	0.3614 (3)	0.0174 (8)	
C7	0.1555 (5)	0.6914 (4)	0.2757 (3)	0.0169 (8)	
H7	0.202597	0.776169	0.260973	0.020*	
C8	−0.1822 (6)	0.3961 (4)	0.1617 (3)	0.0217 (9)	
H8A	−0.273612	0.356019	0.189827	0.026*	
H8B	−0.127352	0.319045	0.134342	0.026*	
C9	−0.2649 (5)	0.4817 (4)	0.0868 (3)	0.0211 (9)	
H9	−0.269614	0.449717	0.024284	0.025*	
C10	−0.3329 (5)	0.6041 (5)	0.1073 (3)	0.0245 (10)	
H10A	−0.329033	0.637354	0.169486	0.029*	
H10B	−0.383691	0.655430	0.059143	0.029*	
O11	0.2235 (4)	0.4935 (3)	0.47161 (19)	0.0224 (7)	
C12	0.1579 (6)	0.3684 (4)	0.4996 (3)	0.0239 (9)	
H12A	0.211332	0.364448	0.564733	0.036*	
H12B	0.186894	0.288719	0.462377	0.036*	
H12C	0.030620	0.366276	0.490730	0.036*	
O13	0.3539 (4)	0.7181 (3)	0.43083 (18)	0.0200 (6)	
C14	0.4376 (6)	0.8428 (4)	0.4126 (3)	0.0219 (9)	
H14A	0.505769	0.893802	0.471514	0.026*	
H14B	0.347315	0.901710	0.384927	0.026*	
C15	0.5587 (6)	0.8167 (4)	0.3483 (3)	0.0239 (10)	
O16	0.5679 (4)	0.7092 (3)	0.3051 (2)	0.0245 (7)	
O17	0.6589 (5)	0.9325 (3)	0.3507 (3)	0.0485 (10)	
C18	0.7874 (8)	0.9294 (6)	0.2922 (5)	0.0561 (14)	
H18	0.823471	0.833482	0.284121	0.067*	
C19	0.9440 (9)	1.0257 (7)	0.3460 (7)	0.091 (2)	
H19A	0.999568	0.985788	0.401411	0.136*	
H19B	0.905267	1.115713	0.363746	0.136*	
H19C	1.028227	1.037723	0.307526	0.136*	
C20	0.7028 (9)	0.9695 (7)	0.1996 (6)	0.0701 (17)	
H20A	0.678687	1.066797	0.206270	0.105*	
H20B	0.592848	0.911267	0.174277	0.105*	
H20C	0.781548	0.956588	0.157863	0.105*	
Cl21	0.16747 (13)	0.78892 (10)	0.07378 (7)	0.0212 (2)	
N22	−0.2017 (4)	0.6881 (4)	−0.0569 (2)	0.0177 (7)	
C23	−0.1998 (6)	0.8167 (4)	−0.0836 (3)	0.0208 (9)	
H23	−0.146276	0.892910	−0.039469	0.025*	
C24	−0.2727 (5)	0.8411 (4)	−0.1723 (3)	0.0194 (9)	
H24	−0.264964	0.932045	−0.189494	0.023*	
C25	−0.3581 (5)	0.7307 (4)	−0.2368 (3)	0.0152 (8)	
C26	−0.3638 (5)	0.5988 (4)	−0.2095 (3)	0.0160 (8)	
H26	−0.422090	0.521699	−0.251417	0.019*	
C27	−0.2827 (5)	0.5822 (4)	−0.1198 (3)	0.0170 (8)	
H27	−0.284227	0.491574	−0.101825	0.020*	
C28	−0.4300 (5)	0.7530 (4)	−0.3353 (3)	0.0173 (8)	
O29	−0.3773 (4)	0.8497 (3)	−0.3691 (2)	0.0245 (7)	
O30	−0.5521 (4)	0.6547 (3)	−0.3784 (2)	0.0275 (7)	
H30	−0.576378	0.661965	−0.435015	0.041*	

### (η2-2-Allyl-4-methoxy-5-{[(propan-2-yloxy)carbonyl]methoxy}phenyl-κC1)chlorido(pyridine-4-carboxylic acid-κN)platinum(II) (III) Atomic displacement parameters (Å^2^)

**Table:**

	*U*^11^	*U*^22^	*U*^33^	*U*^12^	*U*^13^	*U*^23^
Pt1	0.01412 (10)	0.01776 (10)	0.00990 (10)	−0.00293 (7)	−0.00293 (6)	0.00194 (6)
C2	0.014 (2)	0.020 (2)	0.014 (2)	0.0018 (16)	−0.0022 (16)	0.0023 (16)
C3	0.015 (2)	0.023 (2)	0.015 (2)	−0.0046 (17)	0.0008 (16)	−0.0015 (17)
C4	0.019 (2)	0.022 (2)	0.013 (2)	−0.0014 (17)	0.0005 (16)	0.0046 (16)
C5	0.019 (2)	0.023 (2)	0.0099 (19)	0.0038 (17)	−0.0003 (16)	0.0039 (16)
C6	0.017 (2)	0.020 (2)	0.012 (2)	0.0016 (16)	−0.0024 (16)	−0.0019 (16)
C7	0.017 (2)	0.018 (2)	0.0115 (19)	−0.0026 (16)	−0.0020 (16)	−0.0017 (16)
C8	0.024 (2)	0.026 (2)	0.012 (2)	−0.0062 (18)	−0.0012 (17)	0.0041 (17)
C9	0.016 (2)	0.028 (2)	0.015 (2)	−0.0119 (18)	0.0013 (17)	0.0022 (17)
C10	0.013 (2)	0.040 (3)	0.019 (2)	−0.0039 (19)	0.0005 (17)	0.0070 (19)
O11	0.0249 (16)	0.0260 (16)	0.0124 (15)	−0.0013 (13)	−0.0049 (12)	0.0070 (12)
C12	0.026 (2)	0.026 (2)	0.020 (2)	0.0035 (19)	0.0027 (18)	0.0083 (18)
O13	0.0235 (16)	0.0185 (14)	0.0110 (14)	−0.0055 (12)	−0.0084 (12)	0.0013 (11)
C14	0.026 (2)	0.016 (2)	0.018 (2)	−0.0019 (17)	−0.0063 (18)	−0.0013 (16)
C15	0.018 (2)	0.016 (2)	0.032 (3)	−0.0025 (17)	−0.0063 (18)	0.0036 (18)
O16	0.0247 (17)	0.0199 (15)	0.0252 (17)	−0.0025 (13)	0.0003 (13)	0.0011 (13)
O17	0.036 (2)	0.0189 (17)	0.095 (3)	−0.0076 (15)	0.034 (2)	−0.0052 (18)
C18	0.042 (3)	0.023 (3)	0.111 (4)	−0.009 (2)	0.045 (3)	−0.007 (3)
C19	0.045 (3)	0.049 (4)	0.178 (7)	−0.024 (3)	0.047 (4)	−0.020 (4)
C20	0.065 (4)	0.047 (4)	0.125 (4)	0.018 (3)	0.067 (3)	0.023 (3)
Cl21	0.0205 (5)	0.0226 (5)	0.0168 (5)	−0.0075 (4)	−0.0013 (4)	0.0031 (4)
N22	0.0149 (18)	0.0186 (17)	0.0158 (18)	−0.0037 (14)	−0.0022 (14)	0.0006 (14)
C23	0.024 (2)	0.018 (2)	0.016 (2)	−0.0053 (17)	−0.0012 (17)	0.0012 (17)
C24	0.023 (2)	0.0152 (19)	0.016 (2)	−0.0005 (17)	−0.0018 (17)	0.0020 (16)
C25	0.0131 (19)	0.0181 (19)	0.013 (2)	0.0024 (16)	−0.0005 (15)	0.0041 (16)
C26	0.015 (2)	0.0165 (19)	0.013 (2)	−0.0004 (16)	−0.0013 (16)	−0.0014 (16)
C27	0.018 (2)	0.0157 (19)	0.014 (2)	−0.0015 (16)	−0.0020 (16)	0.0026 (16)
C28	0.0174 (18)	0.0186 (15)	0.0125 (18)	0.0013 (12)	−0.0030 (14)	0.0010 (12)
O29	0.0297 (17)	0.0234 (14)	0.0165 (15)	−0.0021 (12)	−0.0035 (13)	0.0067 (11)
O30	0.0302 (17)	0.0320 (15)	0.0113 (15)	−0.0110 (13)	−0.0095 (13)	0.0017 (12)

### (η2-2-Allyl-4-methoxy-5-{[(propan-2-yloxy)carbonyl]methoxy}phenyl-κC1)chlorido(pyridine-4-carboxylic acid-κN)platinum(II) (III) Geometric parameters (Å, º)

**Table:**

Pt1—C2	2.014 (4)	C14—H14B	0.9900
Pt1—C9	2.146 (4)	C14—C15	1.517 (6)
Pt1—C10	2.118 (4)	C15—O16	1.191 (5)
Pt1—Cl21	2.3345 (10)	C15—O17	1.327 (5)
Pt1—N22	2.164 (3)	O17—C18	1.480 (7)
C2—C3	1.396 (6)	C18—H18	1.0000
C2—C7	1.409 (5)	C18—C19	1.517 (8)
C3—C4	1.401 (6)	C18—C20	1.503 (11)
C3—C8	1.502 (5)	C19—H19A	0.9800
C4—H4	0.9500	C19—H19B	0.9800
C4—C5	1.393 (6)	C19—H19C	0.9800
C5—C6	1.401 (6)	C20—H20A	0.9800
C5—O11	1.373 (5)	C20—H20B	0.9800
C6—C7	1.393 (5)	C20—H20C	0.9800
C6—O13	1.390 (5)	N22—C23	1.355 (5)
C7—H7	0.9500	N22—C27	1.346 (5)
C8—H8A	0.9900	C23—H23	0.9500
C8—H8B	0.9900	C23—C24	1.379 (6)
C8—C9	1.523 (6)	C24—H24	0.9500
C9—H9	0.9500	C24—C25	1.396 (5)
C9—C10	1.376 (6)	C25—C26	1.391 (5)
C10—H10A	0.9500	C25—C28	1.504 (5)
C10—H10B	0.9500	C26—H26	0.9500
O11—C12	1.431 (5)	C26—C27	1.385 (5)
C12—H12A	0.9800	C27—H27	0.9500
C12—H12B	0.9800	C28—O29	1.206 (5)
C12—H12C	0.9800	C28—O30	1.318 (5)
O13—C14	1.424 (5)	O30—H30	0.8400
C14—H14A	0.9900		
			
C2—Pt1—C9	81.33 (16)	H12B—C12—H12C	109.5
C2—Pt1—C10	87.62 (17)	C6—O13—C14	118.0 (3)
C2—Pt1—Cl21	93.86 (12)	O13—C14—H14A	109.1
C2—Pt1—N22	176.68 (13)	O13—C14—H14B	109.1
C9—Pt1—Cl21	163.54 (12)	O13—C14—C15	112.5 (3)
C9—Pt1—N22	95.48 (14)	H14A—C14—H14B	107.8
C10—Pt1—C9	37.64 (16)	C15—C14—H14A	109.1
C10—Pt1—Cl21	158.51 (13)	C15—C14—H14B	109.1
C10—Pt1—N22	90.39 (15)	O16—C15—C14	126.1 (4)
N22—Pt1—Cl21	88.97 (9)	O16—C15—O17	125.2 (5)
C3—C2—Pt1	115.9 (3)	O17—C15—C14	108.7 (4)
C3—C2—C7	118.8 (4)	C15—O17—C18	117.4 (4)
C7—C2—Pt1	125.3 (3)	O17—C18—H18	109.1
C2—C3—C4	121.2 (4)	O17—C18—C19	105.4 (5)
C2—C3—C8	117.6 (4)	O17—C18—C20	108.8 (5)
C4—C3—C8	121.0 (4)	C19—C18—H18	109.1
C3—C4—H4	120.2	C20—C18—H18	109.1
C5—C4—C3	119.7 (4)	C20—C18—C19	115.0 (6)
C5—C4—H4	120.2	C18—C19—H19A	109.5
C4—C5—C6	119.5 (4)	C18—C19—H19B	109.5
O11—C5—C4	125.2 (4)	C18—C19—H19C	109.5
O11—C5—C6	115.2 (4)	H19A—C19—H19B	109.5
C7—C6—C5	120.8 (4)	H19A—C19—H19C	109.5
O13—C6—C5	113.7 (3)	H19B—C19—H19C	109.5
O13—C6—C7	125.6 (4)	C18—C20—H20A	109.5
C2—C7—H7	120.0	C18—C20—H20B	109.5
C6—C7—C2	120.0 (4)	C18—C20—H20C	109.5
C6—C7—H7	120.0	H20A—C20—H20B	109.5
C3—C8—H8A	109.7	H20A—C20—H20C	109.5
C3—C8—H8B	109.7	H20B—C20—H20C	109.5
C3—C8—C9	109.9 (3)	C23—N22—Pt1	121.5 (3)
H8A—C8—H8B	108.2	C27—N22—Pt1	120.7 (3)
C9—C8—H8A	109.7	C27—N22—C23	117.8 (3)
C9—C8—H8B	109.7	N22—C23—H23	118.8
Pt1—C9—H9	90.7	N22—C23—C24	122.4 (4)
C8—C9—Pt1	109.2 (3)	C24—C23—H23	118.8
C8—C9—H9	119.1	C23—C24—H24	120.4
C10—C9—Pt1	70.1 (2)	C23—C24—C25	119.3 (4)
C10—C9—C8	121.8 (4)	C25—C24—H24	120.4
C10—C9—H9	119.1	C24—C25—C28	120.4 (4)
Pt1—C10—H10A	107.8	C26—C25—C24	118.6 (4)
Pt1—C10—H10B	89.9	C26—C25—C28	120.8 (4)
C9—C10—Pt1	72.3 (2)	C25—C26—H26	120.7
C9—C10—H10A	120.0	C27—C26—C25	118.6 (4)
C9—C10—H10B	120.0	C27—C26—H26	120.7
H10A—C10—H10B	120.0	N22—C27—C26	123.2 (4)
C5—O11—C12	117.9 (3)	N22—C27—H27	118.4
O11—C12—H12A	109.5	C26—C27—H27	118.4
O11—C12—H12B	109.5	O29—C28—C25	122.4 (4)
O11—C12—H12C	109.5	O29—C28—O30	125.7 (4)
H12A—C12—H12B	109.5	O30—C28—C25	112.0 (3)
H12A—C12—H12C	109.5	C28—O30—H30	109.5
			
Pt1—C2—C3—C4	177.9 (3)	C8—C3—C4—C5	175.9 (4)
Pt1—C2—C3—C8	1.8 (5)	C8—C9—C10—Pt1	100.7 (4)
Pt1—C2—C7—C6	−177.7 (3)	O11—C5—C6—C7	−178.3 (4)
Pt1—N22—C23—C24	179.1 (3)	O11—C5—C6—O13	2.4 (5)
Pt1—N22—C27—C26	178.8 (3)	O13—C6—C7—C2	179.3 (4)
C2—C3—C4—C5	−0.1 (6)	O13—C14—C15—O16	10.9 (6)
C2—C3—C8—C9	−19.4 (5)	O13—C14—C15—O17	−166.5 (4)
C3—C2—C7—C6	0.0 (6)	C14—C15—O17—C18	−179.9 (5)
C3—C4—C5—C6	0.1 (6)	C15—O17—C18—C19	−146.0 (5)
C3—C4—C5—O11	178.1 (4)	C15—O17—C18—C20	90.2 (6)
C3—C8—C9—Pt1	26.1 (4)	O16—C15—O17—C18	2.7 (8)
C3—C8—C9—C10	−51.9 (5)	N22—C23—C24—C25	2.6 (7)
C4—C3—C8—C9	164.5 (4)	C23—N22—C27—C26	−0.2 (6)
C4—C5—C6—C7	−0.1 (6)	C23—C24—C25—C26	−1.2 (6)
C4—C5—C6—O13	−179.5 (4)	C23—C24—C25—C28	−176.6 (4)
C4—C5—O11—C12	1.1 (6)	C24—C25—C26—C27	−0.8 (6)
C5—C6—C7—C2	0.1 (6)	C24—C25—C28—O29	21.8 (6)
C5—C6—O13—C14	176.9 (4)	C24—C25—C28—O30	−159.1 (4)
C6—C5—O11—C12	179.1 (4)	C25—C26—C27—N22	1.6 (6)
C6—O13—C14—C15	−73.7 (4)	C26—C25—C28—O29	−153.5 (4)
C7—C2—C3—C4	0.0 (6)	C26—C25—C28—O30	25.6 (6)
C7—C2—C3—C8	−176.1 (4)	C27—N22—C23—C24	−1.9 (6)
C7—C6—O13—C14	−2.4 (6)	C28—C25—C26—C27	174.6 (4)

### (η2-2-Allyl-4-methoxy-5-{[(propan-2-yloxy)carbonyl]methoxy}phenyl-κC1)chlorido(pyridine-4-carboxylic acid-κN)platinum(II) (III) Hydrogen-bond geometry (Å, º)

**Table:**

*D*—H···*A*	*D*—H	H···*A*	*D*···*A*	*D*—H···*A*
O30—H30···O13^i^	0.84	2.10	2.932 (4)	170
C10—H10*A*···O16^ii^	0.95	2.41	3.317 (5)	159
C12—H12*A*···O16^iii^	0.98	2.51	3.415 (5)	154
C14—H14*A*···O29^iv^	0.99	2.46	3.268 (5)	139
C14—H14*B*···O29^v^	0.99	2.46	3.178 (5)	129
C26—H26···O16^vi^	0.95	2.43	3.336 (5)	159

Symmetry codes: (i) *x*−1, *y*, *z*−1; (ii) *x*−1, *y*, *z*; (iii) −*x*+1, −*y*+1, −*z*+1; (iv) *x*+1, *y*, *z*+1; (v) −*x*, −*y*+2, −*z*; (vi) −*x*, −*y*+1, −*z*.
